# Human intestine and placenta exhibit tissue-specific expression of RAGE isoforms

**DOI:** 10.1016/j.heliyon.2023.e18247

**Published:** 2023-07-18

**Authors:** Katharina Schwertner, Katharina Gelles, Judith Leitner, Peter Steinberger, Claudia Gundacker, Ruben Vrticka, Karin Hoffmann-Sommergruber, Isabella Ellinger, Sabine Geiselhart

**Affiliations:** a Institute of Pathophysiology and Allergy Research, Medical University of Vienna, Vienna, Austria; b Institute of Immunology, Medical University of Vienna, Vienna, Austria

**Keywords:** RAGE, Innate immune receptor, Alternative splicing, Small intestine, Colon, Placenta, Antibody validation

## Abstract

The receptor for advanced glycation end products (RAGE) is encoded by *AGER*, a gene that is subjected to tissue-specific alternative splicing. Splice variants of RAGE in intestine and placenta are unknown and contradictory data concerning RAGE protein expression in these tissues have been published. As a basis for future functional studies, we examined RAGE expression in small intestine, colon and placentas. PCR cloning revealed that full-length RAGE is the only RAGE transcript isoform expressed in placenta. In the small intestine, the major transcript isoform detected was RAGE_v1 encoding the C-terminally truncated soluble receptor. In the colon, both full-length RAGE as well as several splice variants were identified. Four antibodies were used to study protein expression by immunoblotting and were carefully validated. Appropriate controls were essential to avoid misinterpretation of bands caused by non-specific reactivity of antibodies. Only one of four antibodies tested detected full-length RAGE in placenta, whereas no RAGE-specific band was detected in intestinal tissues despite loading >30-fold more intestinal tissue than the positive control, human lung. RAGE expression levels in the placenta were 100-fold lower compared with human lung when analyzed by ELISA, and no significant differences in RAGE expression were detected between healthy placentas and placentas from women with preeclampsia, gestational diabetes mellitus, or fetal growth restriction. We conclude that healthy placental chorionic tissue expresses low levels of full-length RAGE, whereas expression of the tissue-specific intestinal isoforms is below the limit of detection. Low RAGE expression levels in combination with a lack of antibody validation may explain the conflicting published results on RAGE protein expression in intestine and placenta.

## 1 Introduction

The receptor for advanced glycation end products (RAGE) is a multi-ligand binding protein belonging to the immunoglobulin superfamily. Based on its capacity to bind distinct ligands and activate cellular signaling, RAGE is considered as a pattern recognition receptor and classified as innate immune receptor [1]. RAGE was originally cloned from bovine lung and confirmed to bind advanced glycation end products [2]. Since then, many further structurally diverse molecules including S100/calgranulins, high mobility group box 1, and amyloid-β peptides have been identified as ligands [3,4]. RAGE is expressed in several cell types during prenatal and postnatal development, but is downregulated in all tissues except lung after development is complete [5]. However, RAGE can be upregulated again when its ligands accumulate in tissues during aging or disease [6,7]. RAGE ligands are either of endogenous origin and accumulate during aging and inflammation [8] or derive from infections, Western diet or smoking [[9], [10], [11]].

Upon activation, depending on cell type and ligand, RAGE triggers various intracellular signaling cascades leading, e.g., to the production of reactive oxygen species (ROS), immunoinflammatory effects, cell proliferation, or apoptosis [4]. Upregulation of RAGE further enhances RAGE-induced inflammation through positive feedback. Thus, RAGE is a central mediator of immune responses and described to play important roles in a wide range of pathologies such as diabetes mellitus [12], neurological disorders such as Alzheimer's disease [13], atherosclerosis [14], and certain types of cancers [15,16].

Full-length RAGE can structurally be divided into an immunoglobulin variable region including two N-glycosylation sites, two constant immunoglobulin domains, a transmembrane domain, and a cytoplasmic tail. It has a theoretical molecular mass of approximately 48–50 kDa (excluding signal peptide, including two N-glycosylations). However, in SDS-PAGE, full-length RAGE has been shown to migrate with an approximate molecular mass of 55 kDa [[17], [18], [19]]. In addition to the canonical full-length receptor, several splice variants have been described [17,20,21]. Alternative splicing is thought to be a key mechanism determining the expression and function of these isoforms. While up to 50% of annotated RAGE transcripts are subjected to nonsense-mediated mRNA decay (NMD) [17,21], variable usage of exons and introns also contributes to proteomic complexity of the receptor and appears to be important for its diverse physiological functions. Of the known splice variants, RAGE_v1, coding for a soluble version of the receptor lacking the transmembrane as well as the cytoplasmic domain, is the most prominent variant. This isoform, designated endogenous secretory RAGE (esRAGE), presumably acts as a decoy receptor to avoid interaction of full-length RAGE with its ligands [[22], [23], [24]]. Expression of full-length RAGE and splice variants such as RAGE_v1 have been studied in several human tissues such as lung and brain [17,20,25,26]. However, in the intestine and placenta, two important tissues in the context of adult and fetal immune defense, the expression profiles of RAGE isoforms have not yet been studied.

Accumulation of RAGE ligands during chronic inflammation of the gut suggests involvement of RAGE in inflammatory bowel diseases [27]. In the healthy intestine, expression of RAGE was found to be low [28]. It was localized to the surface of the intestinal villi and was confined to the epithelial cell layer [29]. Under inflammatory conditions, however, RAGE expression was increased in inflamed areas of the ileum and colon in epithelial cells as well as in immune cells recruited to the lamina propria [30,31]. Studies in diabetic rats showed a positive staining for RAGE also in crypts and in neurons of the myenteric and submucosal plexus [32]. Taken together, the data are sparse and the role of RAGE in the intestine remains to be fully elucidated.

A contribution of ligand-activated RAGE to pregnancy-associated diseases such as preeclampsia (PE), gestational diabetes mellitus (GDM) and fetal growth restriction (FGR) is suspected, but data confirming this are lacking. In previous reports, RAGE mRNA expression in human placental tissue was either not detected [33] or described as very low [18]. Later studies demonstrated increased levels of RAGE ligands in maternal serum and chorionic placental tissue of pregnancies affected by GDM, PE, and FGR [[34], [35], [36], [37], [38], [39], [40]]. Consequently, further works have focused on comparing RAGE mRNA and protein expression levels in the chorionic villi of healthy and disease-affected placentas. However, the results of these studies differed, with respect to both, the influence of PE, GDM, and FGR on the placental expression levels of RAGE, and the localization of RAGE within the individual cell types of the chorionic villi such as epithelial (trophoblast) cells, endothelial cells or macrophages [37,39,[41], [42], [43], [44], [45]].

Overall, limited and/or controversial published data currently prevent a clear formulation of hypotheses about the role of RAGE in intestine and placenta. It is unknown whether only full-length RAGE, other splice variants or both are expressed in these tissues. Likewise, data on the major protein isoforms expressed in intestine and placenta are lacking. In the latter case, an important factor hindering the acquisition of consistent data could be the quality and specificity of commercial research antibodies [46]. A high degree of non-specificity was recently shown for several commercially available anti-RAGE antibodies [47].

The aims of this study were to characterize RAGE splice variants in human intestine and placenta and to evaluate the major RAGE isoforms of these tissues on the protein level. We performed a detailed analysis of RAGE splice variants expressed in the healthy intestine (small intestine and colon) and placenta, and compared the data to the previously studied tissues lung and brain [17,25]. In addition, puzzled by the varying published results, we reexamined RAGE protein expression in the intestine and placenta by antibody-based methods. For this purpose, we first investigated the specificity of four anti-RAGE antibodies and validated their suitability for Western blotting (WB) and immunofluorescence microscopy (IFM). We then comparatively studied the expression and localization of RAGE in healthy small intestine and colon, as well as in placentas (placental chorionic tissue) derived from healthy and GDM-, PE-, and FGR-impaired pregnancies. Furthermore, we examined RAGE protein expression in intestinal and placental cell lines with the goal of establishing appropriate in vitro systems for functional studies.

## 2 Results

### 2.1 Small intestine, colon and placenta exhibited a differential, tissue-specific expression of RAGE isoforms

**Fig. 1:**
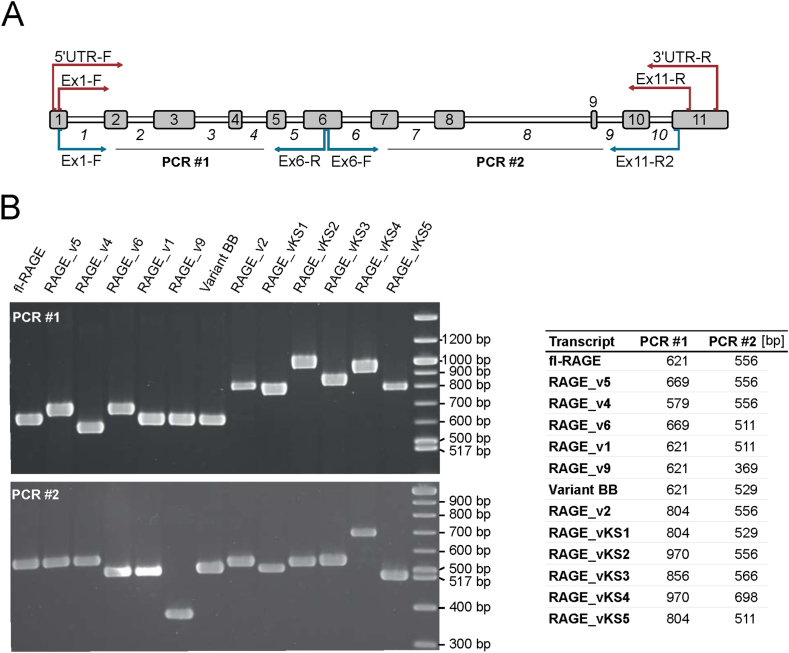
Detection of RAGE transcript variants by PCR. (A) Splice variants were amplified from cDNA by a nested PCR approach using the RAGE-specific primer pairs indicated in red. For variant identification, two independent PCRs were performed on cloned cDNA using the primers marked in blue. (B) Agarose gel electrophoresis of products from PCRs for variant identification showing a specific pattern for each detected variant. PCR product size was calculated from sequencing data and is indicated in the table on the right. (bp) base pairs. The uncropped images are shown in the supplementary material of the manuscript. (For interpretation of the references to color in this figure legend, the reader is referred to the Web version of this article.)

**Fig. 2:**
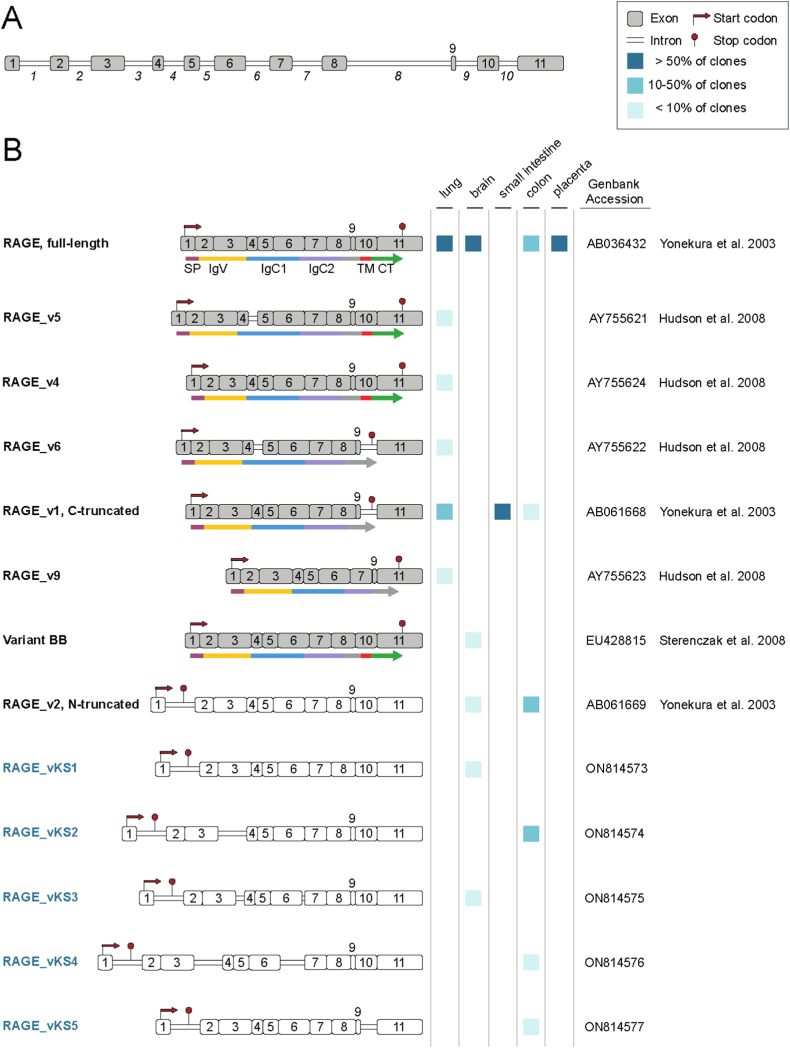
Characterization of RAGE transcript variant variants. (A) The AGER gene consists of 11 exons and 10 introns. (B) AGER encodes the canonical full-length RAGE, a membrane-bound protein of 404 amino acids composed of a signal peptide (SP), a variable immunoglobulin domain (IgV), two constant Ig domains (IgC1 and IgC2) a transmembrane domain (TM) and a cytoplasmic tail (CT). Additionally, alternative splicing generates several transcript isoforms. Individual isoforms are detected in lung, brain, small intestine, colon, and placenta and show tissue-specific expression patterns. Potential proteins of coding variants are depicted in grey, while non-coding RNAs are shown in white. Names of newly identified variants are indicated in blue. (For interpretation of the references to color in this figure legend, the reader is referred to the Web version of this article.)

In contrast to human lung and brain where RAGE splice variants have already been studied, their individual occurrence and abundance in human small intestine and colon as well as placenta was unknown. Thus, commercially available RNA samples representing pools of healthy tissues (i.e., lung, brain, small intestine, colon, and placenta) were investigated by PCR cloning. For each tissue, over 30 clones were analyzed and transcript variants were identified by a combination of PCR and subsequent sequencing as outlined in Fig. 1A. In total, we identified thirteen RAGE transcript variants, five of which (RAGE_vKS1-5) are described for the first time (Fig. 1B). Tissue-specific expression patterns of these variants are shown in Fig. 2.

Full-length RAGE, encoding the canonical transmembrane receptor, was the most abundant isoform in lung, brain and placenta; it was also a major isoform in colon (Fig. 2B). Of interest, in placenta, only full-length RAGE and no other splice variant was observed. Another interesting finding was the lack of full-length RAGE in human small intestine. The only transcript isoform identified in small intestine was RAGE_v1 encoding the C-terminally truncated secretory receptor esRAGE. RAGE_v1 was also found in colon and in lung; in the latter, it was the second most abundant isoform.

Additional RAGE transcript variants were identified in lung, colon and brain. Our analysis of human lung revealed transcript variants with partial retention of intron 4 (RAGE_v5 and RAGE_v6) and isoforms with partial deletion of exon 3 (RAGE_v4) or lack of exon 8 (RAGE_v9). In brain, variant BB lacking exon 9 was identified in addition to full-length RAGE. Retention of intron 1, likely resulting in a premature stop codon within exon 1, was frequently observed in brain and colon (e.g., in RAGE_v2). Intron 1 was also maintained in all novel transcript isoforms: RAGE_vKS1 and RAGE_vKS3 were isolated from brain with RAGE_vKS1 additionally lacking exon 9 and RAGE_vKS3 containing parts of intron 3 and 6. RAGE_vKS2, expressed in human colon, shows complete retention of introns 1 and 3, while RAGE_vKS4 shows complete retention of introns 1, 3, and 6. Furthermore, RAGE_vKS5, combining splicing events of RAGE_v1 and RAGE_v2, was discovered in colon and exhibits retention of intron 1 and part of intron 9, skipping exon 10. In total we identified thirteen RAGE transcript variants, five of which (RAGE_vKS1-5) were described for the first time.

### 2.2 High RAGE mRNA levels are only detected in the lungs

In contrast to the high mRNA expression in lung, low to undetectable expression of RAGE in healthy small intestine, colon and placenta as well as brain had been reported [18,33,48]. To address and compare total RAGE mRNA levels of brain, small intestine, colon, and placenta with expression levels in the lung, we used the GENEVESTIGATOR platform (NEBION AG, Zurich, Switzerland) that comprises curated transcriptomic data from numerous public repositories [49,50]. Both datasets, from a microarray and an RNAseq platform, confirmed large differences in expression levels between lung and the other tissues (Figure S1, A and B, respectively).

### 2.3 Three commercial anti-RAGE antibodies detected the two major RAGE isoforms, full length RAGE and esRAGE, in human lung tissue by WB

**Fig. 3:**
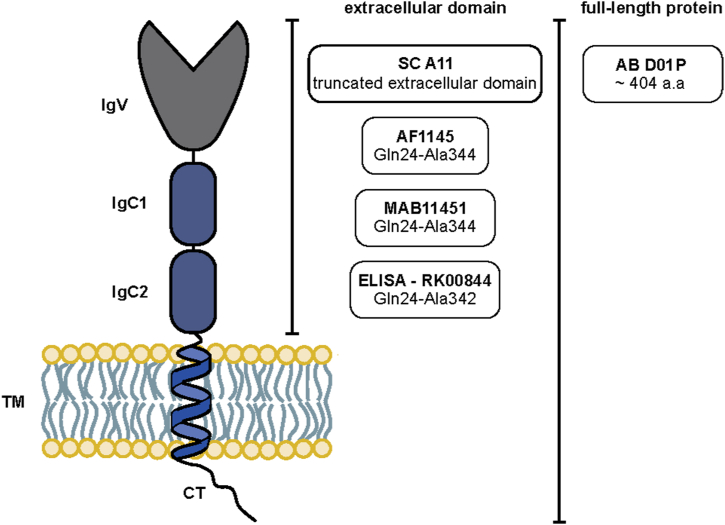
Schematic representation indicating immunogenic sequences within human full-length RAGE protein of the anti-RAGE antibodies tested in this study. Indicated domains: signal peptide (SP), variable immunoglobulin domain (IgV), two constant Ig domains (IgC1 and IgC2), transmembrane domain (TM) and cytoplasmic tail (CT).

To reveal the major RAGE isoforms expressed in small intestine, colon, and placenta, we applied several antibody-based methods (i.e., WB, IFM and enzyme-linked immunosorbent assay (ELISA). The immunogens of all antibodies employed in this study are detailed in Fig. 3.

For WB experiments, we tested four commercially available RAGE antibodies (SC A11, AF1145, AB D01P, and MAB11451) following current suggestions for antibody validation [51]. We first evaluated the antibodies on human lung lysate, which exhibits the highest RAGE expression levels among all adult tissues [5]. As shown in previous studies [17,20,26] and confirmed in our transcript analysis, the most abundant isoforms in lung are full-length RAGE and RAGE_v1, which is translated to esRAGE.

**Fig. 4:**
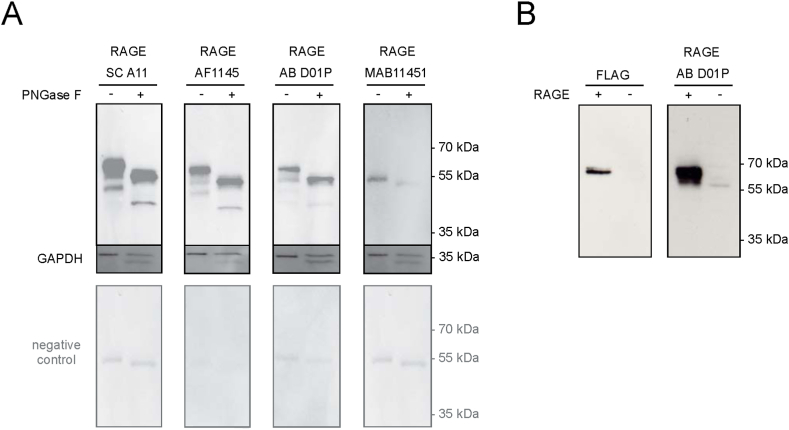
(A) RAGE immunoblotting analysis of human lung lysate. SDS-PAGE of lung lysate (1 μg/lane; PNGase F -) and deglycosylated lung lysate (1 μg/lane; PNGase F +) was performed under reducing conditions. Membranes were stained with four different anti-RAGE antibodies. Membranes were also stained with the respective secondary antibodies only (negative control). GAPDH was used as a loading control. (B) RAGE immunoblotting analysis of RAGE overexpressing (+) and control (−) HEK293T cells. SDS-PAGE was performed under reducing conditions. For WB analysis membranes were stained with anti-FLAG and anti-RAGE (AB D01P) antibodies. The uncropped images are shown in the supplementary material of the manuscript.

Three of the four tested antibodies (SC A11, AF1145, and AB D01P) recognized two bands at approximately 58 and 45 kDa in commercially available lung lysates (Fig. 4A, upper panel). When we used MAB11451, these bands were not visible. All four antibodies recognized a faint additional band at around 53 kDa. However, this band was also detected when membranes were probed with the respective secondary antibodies only (Fig. 4A; lower panel) and was therefore considered as unspecific.

To confirm that the band at approximately 58 kDa was in fact full-length RAGE, we performed a WB using commercially available lysates from HEK293T cells overexpressing full-length RAGE additionally containing a Myc/FLAG-tag (Fig. 4B). Lysate of mock-transfected HEK293T cells was used as a negative control. When probing the membrane with an anti-FLAG antibody, a band slightly above 60 kDa became visible, whereas no signal could be observed when using lysate from mock-transfected cells (Fig. 4B, left panel). The RAGE-specific antibody AB D01P (Fig. 4B, right panel) detected a band of identical size, confirming that the protein detected is indeed RAGE. The slight increase in size of full-length RAGE compared to Fig. 4A (∼60 versus 58 kDa) is due to the additional amino acids of the Myc/FLAG-tag. The faint band at around 58 kDa in the control lane corresponds to the low level of endogenous RAGE expression described in HEK293T cells [49].

To determine whether the proteins at 58 and 45 kDa contain N-linked glycans, we subjected lung lysate to enzymatic treatment with Peptide N Glycosidase F (PNGase F). Both bands shifted and two bands of approximately 53 and 40 kDa appeared corresponding to two deglycosylated RAGE isoforms (Fig. 4A). Interestingly, the third band visible with the secondary antibodies also shifted, suggesting that this is also a glycoprotein. Repetition of these experiments gave identical results (Figure S2).

Since full-length RAGE and variant RAGE_v1 are the most abundant transcripts in lung, we concluded that the bands at 58 and 45 kDa observed in human lung tissue correspond to glycosylated full-length RAGE and the C-truncated variant esRAGE, respectively.

### 2.4 The low endogenous RAGE expression rate in intestine and placenta made it difficult to determine its tissue-specific isoforms at the protein level

**Fig. 5:**
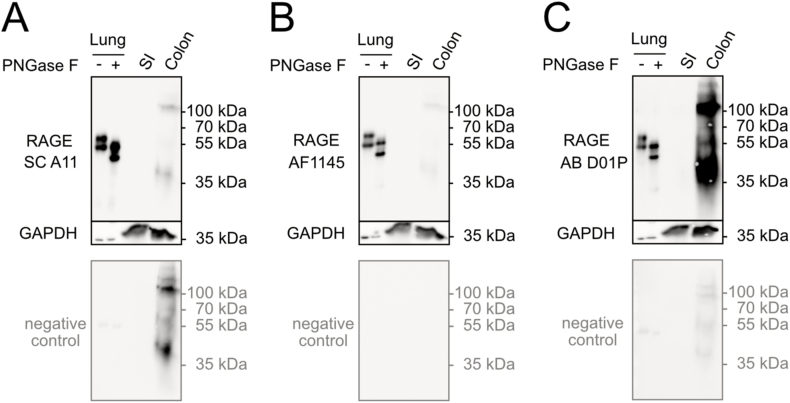
RAGE immunoblotting analysis of human intestine. SDS-PAGE of lung lysate (1.5 μg/lane) as well as lysates from small intestine (SI) and colon (50 μg/lane) was performed under reducing conditions. Membranes were stained with three different anti-RAGE antibodies; SC A11 (A), AF1145 (B), and AB D01P (C). Membranes were also stained with the respective secondary antibodies only (negative control). GAPDH was used as a loading control. The uncropped images are shown in the supplementary material of the manuscript.

Next, we addressed RAGE protein expression in intestinal and placental tissue samples. In contrast to the high mRNA expression in lung, low-to-undetectable expression of RAGE in healthy small intestine, colon and placenta as well as brain had been reported [18,33,48]. Thus, much higher amounts of intestinal and placental tissue lysates were used in comparison to lung tissue lysate which was used as a positive control for WB (Fig. 5 A-C and Fiig. 6 A-C).

None of the antibodies showed RAGE-specific staining for small intestine (Fig. 5A–C). In colon lysate all three antibodies showed a signal at approximately 110 kDa of varying strength (Fig. 5A–C) and two of the antibodies (SC A11 and AB D01P) showed several additional signals of different sizes (Fig. 5 A and C). However, when probing the membranes with the respective secondary antibody only, these signals were also visible. It cannot be excluded that some of the signals are RAGE-specific. However, the result is difficult to interpret due to the strong unspecific background staining in colon.

**Fig. 6:**
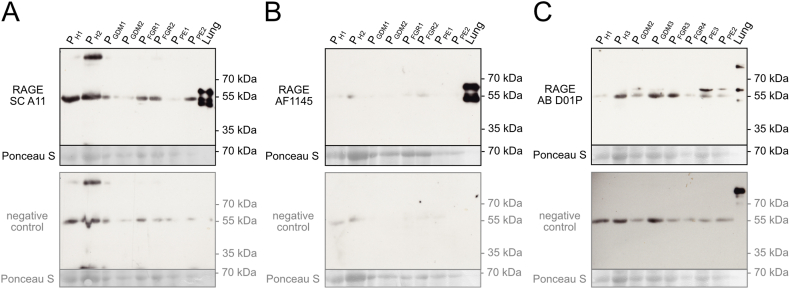
RAGE immunoblotting analysis of human placenta derived from different healthy (H) as well as GDM-, FGR- and PE-affected pregnancies. SDS-PAGE of lung lysate (1 μg/lane) as well as lysates from the placentas (25 μg/lane) was performed under reducing conditions. For WB analysis membranes were stained with three different anti-RAGE antibodies; SC A11 (A), AF1145 (B), and AB D01P (C). Membranes were also stained with the respective secondary antibodies only (negative control). Ponceau S staining was used as a loading control. The uncropped images are shown in the supplementary material of the manuscript.

Results for RAGE expression in human healthy placenta are shown in Fig. 6 A-C. Since several studies have suggested altered RAGE protein expression levels due to pregnancy diseases, we included lysates derived from PE-, GDM-, and FGR-affected placentas. In comparison to human lung, where full-length RAGE and esRAGE could be clearly detected despite the low amount of protein loaded, the antibodies SC A11, AF1145, and AB D01P predominantly detected a band of 53 kDa in placental samples (Fig. 6A–C). Although this protein is in the range of the estimated molecular mass of RAGE, the signal is not related to RAGE, as revealed by probing the same samples with secondary antibodies only. The molecular mass of the protein corresponds to the non-RAGE protein also detected in lung lysate (53 kDa). Anti-RAGE antibody AB D01P was the only antibody that in some placental samples detected a band corresponding to the full-length RAGE (58 kDa) also seen in human lung positive controls (Fig. 6C and Figure S3). This protein band was not detected with secondary antibodies alone and thus may represent full-length RAGE.

As an independent proof for low RAGE expression in different placental tissue samples, we analyzed three different placenta samples per group with a RAGE-specific commercial ELISA. In comparison to human lung, human healthy placentas, PE-, GDM-, and FGR-affected placentas exhibited low expression levels of RAGE (Table 1). Statistical analysis revealed no significant differences in RAGE expression levels between placentas derived from healthy and GDM-, PE- and FGR-affected pregnancies (Kruskal-Wallis H test, χ^2^(3) = 2.538, *p* = 0.468). Thus, the results of the ELISA assay confirmed low RAGE protein expression in placental samples, but could not detect any of the purported dependence of this expression on disease state.

**Table 1:** Human RAGE concentration in lung and placental chorionic tissue samples determined with a RAGE-specific ELISA kit (ABclonal RK00844).

Tissue (human)	Samples	*pg RAGE/mg protein*	*Mean pg RAGE/mg protein*	*SD pg RAGE/mg protein*
**Lung (n** = **1)**	L1		11 448	nd
**Placenta (healthy)** **(n** = **3)**	H1	171.1	106	57
	H2	82.6		
	H3	65.7		
**Placenta (GDM)** **(n** = **3)**	GDM1	33.8	75	43
	GDM2	118.7		
	GDM3	72.2		
**Placenta (PE)** **(n** = **3)**	PE1	28.5	120	82
	PE2	147.7		
	PE3	184.7		
**Placenta (FGR)** **(n** = **3)**	FGR1	22.8	52	28
	FGR2	55.6		
	FGR3	78.4		

GDM, gestational diabetes mellitus; PE, preeclampsia; FGR, fetal growth restriction.

**Fig. 7:**
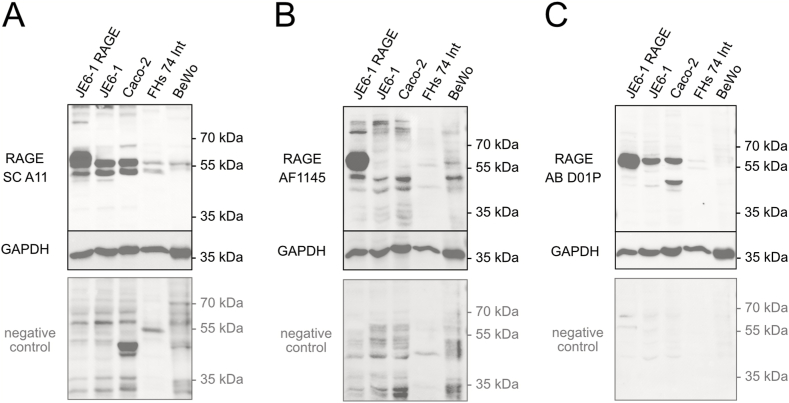
RAGE immunoblotting analysis of human epithelial cell lines. SDS-PAGE of JE6-1 RAGE, JE6-1, Caco-2, FHs 74 Int and BeWo cells (25 μg/lane) was performed under reducing conditions. For WB analysis membranes were stained with three different anti-RAGE antibodies; SC A11 (A), AF1145 (B), and AB D01P (C). To identify unspecific binding, membranes were also stained with the respective secondary antibodies only (negative control). GAPDH staining was used as a loading control. The uncropped images are shown in the supplementary material of the manuscript.

In order to evaluate their usability to serve as in vitro models, we investigated RAGE protein expression in intestinal (Caco-2 and FHs 74 Int) and placental (BeWo) cell lines. Here, the full-length RAGE overexpressing cell line (JE6-1 RAGE) was used as positive control, where most antibodies predominantly detected a band at 58 kDa (Fig. 7). In all cell lines with expected low endogenous RAGE expression, i.e., untransfected JE6-1 cells, the intestine-derived cell lines Caco-2 and FHs 74 Int and the placental cell line BeWo, the WB analysis shown in Fig. 7 revealed that the different RAGE-specific antibodies recognize distinct patterns of bands. RAGE-specific bands could be detected at approximately 58 kDa with the AB DP01 antibody (Fig. 7C) or 58 kDa and 53 kDa when using the SC A11 antibody (Fig. 7A). With AF1145, the band at 58 kDa was only detected in lysates from RAGE overexpressing JE6-1 cells, but not in other cell lines (Fig. 7B). Furthermore, it became apparent that RAGE expression in FHs 74 Int and BeWo cells is significantly lower than in JE6-1 and Caco-2 cells.

### 2.5 Detection of full-length RAGE by IFM depended on the expression level

**Fig. 8:**
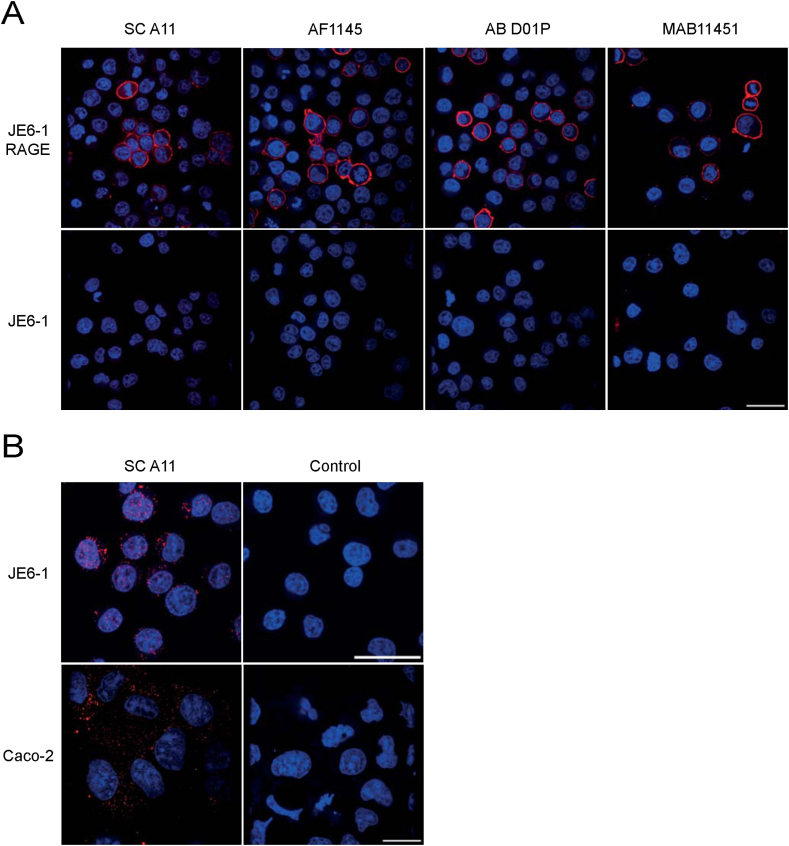
RAGE subcellular localization in human cell lines. (A) JE6-1 cells overexpressing full-length RAGE as well as wild-type control cells were stained with four different RAGE-specific antibodies (SC A11, AF1145, AB D01P and MAB11451). Nuclei were stained with DRAQ5. Scale bar: 25 μm. (B) RAGE staining of JE6-1 cells and intestinal epithelial cells Caco-2 using antibody SC A11 and prolonged exposure times. Controls were stained with the respective secondary antibody only. Nuclei were stained with DRAQ5. Scale bar: 25 μm.

We also evaluated usability of the four anti-RAGE antibodies (SC A11, AF1145, AB D01P, and MAB11451) in IFM experiments as a prerequisite to further explore cellular and subcellular RAGE localization. JE6-1 cells overexpressing the transmembrane containing full-length variant of RAGE (JE6-1 RAGE) were used in the validation experiments (Fig. 8A). All antibodies tested resulted in a strong signal on the cell surface as expected for full-length RAGE (Fig. 8A, upper panel), whereas untransfected control cells (JE6-1) did not show any staining under identical labelling and acquisition conditions (Fig. 8A, lower panel).

When higher antibody concentrations and increased exposure times were used, a positive staining was also observed in untransfected JE6-1 cells (Fig. 8B, left upper panel). These signals, however, were not located at the plasma membrane, but distributed in the cytoplasm showing a dispersed punctuate pattern. A similar pattern became visible in the widely used colon carcinoma cell line Caco-2 (Fig. 8B, left lower panel) that showed expression levels comparable to JE6-1 cells. Positive signals could be identified mainly within the cytoplasm showing a vesicular pattern throughout the cell. Control stainings lacking the primary antibody were negative (Fig. 8B, right panels). Similar results could be obtained when using the other RAGE-specific antibodies (data not shown).

**Fig. 9:**
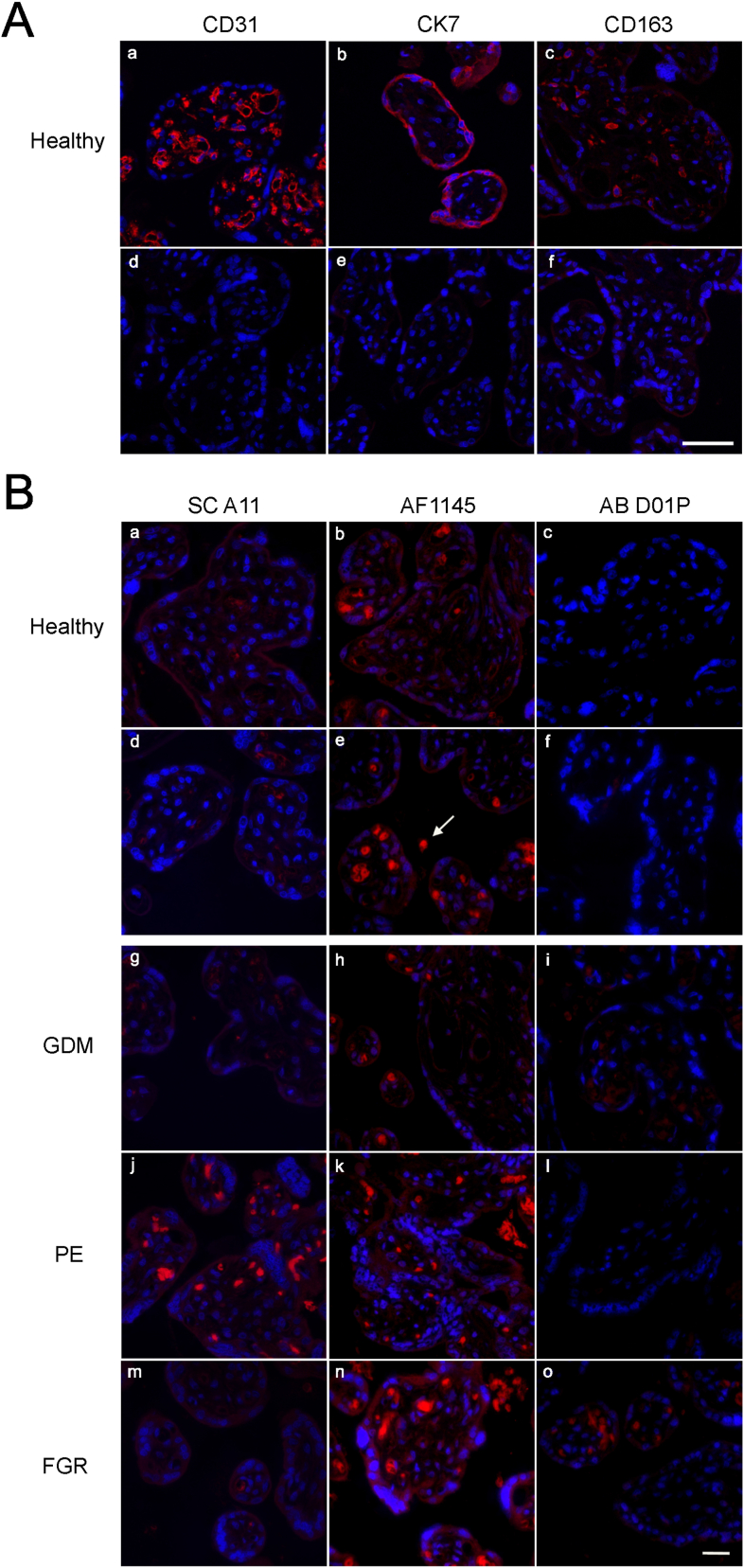
Immunofluorescence staining of human placental sections. Nuclei were stained with DRAQ5 (A) Staining of healthy placental tissue sections. Endothelial cells were stained with CD31 (a), epithelial cells (trophoblasts) were stained with CK7 (b) and macrophages (Hofbauer-cells) were stained with CD163 (c). Respective negative controls are shown in d-f. Scale bar is 50 μm. (B) Staining of healthy and diseased placental tissue sections. Three different RAGE antibodies (SC A11, AF1145, and AB D01P) were used. None of these antibodies resulted in a specific staining. The white arrow indicates one of many erythrocytes within fetal vessels or the intervillous space that exhibit high levels of autofluorescence. Respective negative controls for a-c are shown in d-f. Scale bar is 20 μm.

Since we could detect full-length RAGE protein in some of the WB experiments (Fig. 6 and Figure S3), we subsequently also investigated RAGE localization in placental tissue samples by IFM. To better understand which cell types at the placental barrier may express RAGE, we first identified endothelial (placental-fetal endothelial cells), epithelial (trophoblast cells), and macrophage cells (Hofbauer cells) in sections of chorionic villi with the specific marker proteins CD31, cytokeratin 7 (CK7), and CD163, respectively (Fig. 9A, a-c). Control stainings lacking the primary antibody were negative (Fig. 9A, d-f). We then performed experiments applying three different anti-RAGE antibodies (SC A11, AB D01P, and AF1145) on sections of three placentas per group (healthy, GDM, PE, FGR). Apart from intense fluorescence signals caused by autofluorescence of erythrocytes trapped within fetal vessels in the chorionic tissue (Fig. 9B), sections incubated with primary anti-RAGE and fluorochrome-conjugated secondary antibodies (Fig. 9B, a-c) were indistinguishable from the negative control stainings lacking the primary antibodies (Fig. 9B, d-f). Furthermore, placental sections derived from diseased placentas (Fig. 9B, g-o) were indistinguishable from healthy sections (Fig. 9B, a-c).

In summary, consistent with the WB experiments, detection of RAGE by IFM depended on the overall level of RAGE expression in the tissue or cell line examined. It could be accomplished in a cell line with high expression of RAGE due to cDNA transfection as well as in JE6-1 and Caco-2 cells, but was not successful in placental tissue exhibiting low endogenous expression levels.

## 3 Discussion

RAGE is subject of alternative splicing and several isoforms have been described. The main isoforms detected in most studies are full-length RAGE (RAGE) and the C-truncated soluble variant esRAGE encoded by RAGE_v1 [17,25,26]. The latter variant was first described in 2003 by Yonekura et al. containing part of intron 9 but lacking the exon 10 sequence encoding the transmembrane domain [19]. We analyzed commercially available RNA samples representing pools of healthy tissues from human small intestine, colon, and placenta for the expression of RAGE isoforms in comparison to human lung and brain tissue that had been previously investigated [17,20,25,26]. In these tissues, we identified thirteen RAGE transcript variants, five of which (RAGE_vKS1-5) were described for the first time.

The major variants we detected in the lung were full-length RAGE and RAGE_v1. We could further isolate variants RAGE_v5, RAGE_v4, RAGE_v6 and RAGE_v9. All these variants had already been described by Hudson et al. (2008) [17]. RAGE_v5, RAGE_v4 and RAGE_v6 show changes in the extracellular domain. RAGE_v9 is characterized by deletion of exon 8 and was previously isolated from human primary aortic smooth muscle cells. Most of the transcript variants detected in lung so far are protein-coding and, with the exception of RAGE_v1, they possibly increase versatility and specificity of RAGE by generating isoforms with changes in the ligand binding performance of the receptor as well as its multimerization behavior [52].

In brain tissue, full-length RAGE was the most prevalent variant. This is in accordance with previous data from Jules et al. (2013) [26] reporting 90% abundance of full-length RAGE, whereas other investigators found 4-fold higher soluble RAGE levels than the full-length transcript [20,25]. We could not isolate RAGE_v1 from brain RNA at all. Instead, we found low expression of two other variants that have been described previously – RAGE_v2, an N-truncated variant containing intron 1 [17,19] and variant BB lacking exon 9 [53]. Additionally, two new intron 1-containing variants were isolated (RAGE_vKS1 and 3).

In healthy placenta, full-length RAGE was the only variant that could be isolated within this study. In the colon, full-length RAGE was the most prevalent isoform, accounting for approximately 50% of the transcripts and only low levels of RAGE_v1 were detected. Additionally, three new intron 1-containing variants were isolated (RAGE_vKS2, 4 and 5). However, retention of intron 1 results in a premature termination codon targeting the transcripts to NMD, an important cellular control mechanism. Recent studies, however, have also shown a role in regulating gene expression through NMD [54]. The increased abundance of intron 1-containing transcripts in certain tissues such as brain and colon could thus be an indication for a regulatory function of these transcripts. RAGE_v1 coding for esRAGE was the sole transcript variant isolated from small intestinal RNA. In most investigated tissues, the levels of esRAGE remain low compared to full-length RAGE [17,20,26]. The formation of soluble receptor variants is a well-known phenomenon offering a mechanism to regulate immune responses [55]. Such soluble variants are usually secreted to antagonize the biological activity of the full-length receptor, thus balancing the immune reactions [55]. For soluble RAGE it was shown that besides functioning as a decoy receptor, it can bind to the leukocyte integrin MAC-1, triggering pro-inflammatory signals and leukocyte recruitment [23]. In line with this, both decreased and increased serum levels of soluble RAGE are associated with a range of pathologies [56,57]. In any way, the ratio of full-length RAGE/soluble RAGE seems to be a powerful mechanism to fine-tune RAGE signaling [[58], [59], [60]].

In adult tissues, except in lung, RAGE is constitutively expressed at very low levels under physiological conditions [5], but RAGE expression can be upregulated during certain disease states [5,61]. Strong evidence implicates RAGE in the pathophysiology of lung diseases [62] or diabetic complications [63]. In contrast, information on key aspects of this important innate immune receptor remains sparse in the placenta and intestine, where an involvement of RAGE in the pathogenesis of pregnancy-associated diseases (PE, GDM, FGR) [39,41,43,[64], [65], [66]] and inflammatory bowel disease [[30], [31], [32],[67], [68], [69]] has been suspected but remains controversial.

High mRNA expression in lung, but low-to-undetectable expression of RAGE in healthy small intestine, colon, placenta and brain had been reported [18,33,48]. Our study of publicly available databases using Genevestigator (Figure S1) as well as data of the Human Protein Atlas confirm these expression levels [49,50]. The results of WB experiments determining protein expression in lung, intestine and placenta are in line with these studies. In the lung, strong signals even with low amount of loaded protein were obtained in WB analysis. Immunoblotting revealed two RAGE-specific bands at approximately 58 and 45 kDa that, upon PNGase F treatment, shifted resulting in two bands of approximately 53 and 40 kDa (Fig. 4 and S2). Together with what is known from previous studies, these two isoforms most likely correspond to glycosylated full-length RAGE and the endogenous secretory isoform esRAGE [17,19]. This correlates well with the two major transcripts detected in the lung. A third faint band at around 53 kDa was also visible due to reaction of the secondary antibodies. The slight shift after PNGase F treatment let us speculate that this is due to binding of the secondary antibody to the heavy chain of IgG present in tissue lysates that has a molecular mass of about 50 kDa and contains at least one N-linked carbohydrate [70].

Whereas in lysates from small intestine RAGE protein levels remained below the detection limit of the WB experiments, in lysates from colon tissue, several bands of different sizes appeared. However, probing the membranes with the secondary antibody only, resulted in a similar pattern, leading to the conclusion that most of these signals are unspecific and RAGE protein levels in total tissue lysates of healthy intestine are below the detection limit of the WB system. We cannot exclude that same of the signals are RAGE-specific, but the WB results are hard to interpret due to the strong background in this tissue. Body-Malapel et al., 2018, had quantified RAGE expression in human colon tissue using an ELISA system and found very low RAGE expression in healthy human colon tissue, which is in line with our results.^30^ But further published results are quite contradictory: whereas some authors were able to detect RAGE by WB in normal colonic tissue adjacent to tumors [71], Kuniyasu et al. even reported absence of RAGE mRNA from normal colon mucosa [72]. In contrast to the tissue lysates, where we could not clearly detect RAGE protein, full-length RAGE was the major isoform in Caco-2 cells as demonstrated with two antibodies (SC A11, and AB D01P). RAGE expression in CaCo-2 cells that are a human colon adenocarcinoma cell line has been reported [73] and higher expression levels of RAGE in colon cancer cells is in line with a study from Sakellariou et al. [71]. Information concerning the localization of RAGE in the human intestine is sparse and inconsistent. According to Zen et al. (2007), RAGE is located at intestinal epithelial lateral membranes close to the apical cell junction complexes [74]. Others showed that RAGE is present in the epithelial cells of the villi mainly at the tip, homogeneously distributed in the cell membrane whereas in crypt regions it is located in the cytoplasm [32]. Immunofluorescence analysis of the epithelial cell line Caco-2 revealed RAGE staining mainly in granular structures distributed throughout the cell. In untransfected JE6-1 cells, with low endogenous full-length RAGE expression the protein was also localized mainly in the cytoplasm (Fig. 6). Only full-length RAGE-transfected JE6-1 cells with significantly increased RAGE protein levels, exhibited bright membrane staining. RAGE transport to the cell membrane and subsequent intracellular targeting depends on various signals that are currently not fully understood [75,76]. Intracellular RAGE localization has also been shown in other studies [77,78], but the sorting signals and mechanisms leading to different intracellular localizations are not known.

We could only detect very low amounts of RAGE protein in placental tissues derived from healthy as well as disease-affected pregnancies (PE, FGR, GDM) compared to human lung using a commercial ELISA assay. In line with these results, RAGE protein expression levels were also not detected by two of the tested RAGE-specific antibodies (SC A11, AF1145) in WB and IFM experiments. One antibody (AB D01P) detected faint bands at 58 kDa in some samples by WB, but not in IFM experiments. The size of the protein observed in the WB experiments is consistent with the fact that full-length RAGE was the only placental transcript we could detect. Our data demonstrated low protein expression in healthy placentas, which is in line with the observation of low-to-undetectable RAGE expression in human placenta in several studies [18,33,48]. RAGE expression in the placenta-derived cell line BeWo was also found to be low, which is in line with the low RAGE expression observed by others [43]. In contrast to our results of unchanged RAGE expression in placental tissue derived from disease-affected pregnancies (PE, FGR, GDM), several published studies had reported changes of placental RAGE expression in PE, GDM and FGR-affected pregnancies [37,39,[41], [42], [43],[64], [65], [66]]. However, again, published studies are contradictory as studies showing e.g., increased RAGE expression in PE-affected placentas [37,41,66] contrast with others that found no changes in RAGE expression levels [39,64].

Overall, we could show tissue-specific expression of RAGE transcript isoforms in small intestine, colon, and placenta. The predominant isoforms were full-length RAGE, found in colon and placenta as well as RAGE_v1 detected in small intestine and colon. Moreover, we identified five novel RAGE transcript variants from brain and colon, all containing intron 1 and thus directed to NMD. We validated four anti-RAGE antibodies by WB and IFM and examined RAGE expression in placentas from healthy and diseased pregnancies, in the intestine, and in intestinal and placental epithelial cell lines; these tissues and cell lines had very low RAGE expression, which made detection of the protein challenging, particularly because WBs often contained signals that were not associated with RAGE and could only be identified with the inclusion of appropriate controls. Since the expected molecular mass of full-length RAGE is approximately 55 kDa [[17], [18], [19]], signals detected at this size might easily be misinterpreted as RAGE-specific, especially if an antibody is recommended for WB and this is the only signal obtained (see Fig. 4).

We speculate that the conflicting published results on RAGE protein expression in intestine and placenta may be related to the low endogenous RAGE expression in combination with the fact that the different anti-RAGE antibodies applied recognize different patterns of protein bands, an issue that has been described for other proteins as well [79]. The lack of antibody validation in the vast majority of studies may also contribute to inconsistent data concerning RAGE protein expression and localization.

Our study clearly shows that apart from a detailed and tissue-specific analysis of RAGE isoforms, antibody validation and method-specific optimization is inevitable to study RAGE, especially when tissues with low RAGE expression such as intestine and placenta are analyzed. When interpreting immunoblots, it is important to keep in mind that post-translational modifications of RAGE can lead to changes in the molecular mass of the protein and that these can also differ among different tissues and cell lines. Moreover, some of these post-translational modifications might be recognized differently by individual antibodies. Apart from glycosylation, other post-translational modifications may exist that could impact the molecular mass, ligand binding, antibody recognition and signaling [[80], [81], [82], [83]]. To achieve substantial progress in understanding the biological roles of RAGE, precise analysis of RAGE isoforms in individual tissues and different diseases appears mandatory. Detailed knowledge of RAGE expression, intracellular processing, post-translational modifications as well as careful interpretation of the obtained data is a prerequisite to get a better understanding of this highly complex system.

## 4 Methods

### 4.1 Chemicals and reagents

Antibodies against human RAGE (Fig. 3) were purchased from R&D Systems (AF1145; goat polyclonal IgG antibody raised against mouse myeloma cell line NS0-derived recombinant human RAGE Gln24-Ala344 and MAB11451; clone #176902; mouse monoclonal IgG2b antibody raised against mouse myeloma cell line NS0-derived recombinant human RAGE Gln24-Ala344), Santa Cruz (sc-80652; clone A11; mouse monoclonal IgG2a antibody raised against a truncated extracellular domain of RAGE of human origin) and Abnova (H00000177-D01P; rabbit polyclonal IgG antibody raised against a full-length human RAGE protein). All antibodies are recommended for WB and two antibodies (SC A11, AF1145) are also suggested for IFM. The mouse monoclonal anti-FLAG M2 antibody was purchased from Sigma-Aldrich. Mouse anti-human CD163 was obtained from Invitrogen and mouse anti-human CK7 and the mouse anti-human CD31 were purchased from Dako. The antibody for GAPDH was obtained from Abcam (rabbit monoclonal IgG). HRP-linked secondary antibodies were obtained from Abcam (rabbit anti-mouse IgG; goat anti-rabbit IgG; and Santa Cruz (donkey anti-goat IgG). Alexa Fluor™ 568 labelled secondary antibodies (goat anti-mouse IgG (H + L) cross-adsorbed secondary antibody; goat anti-rabbit IgG (H + L) cross-adsorbed secondary antibody; donkey anti-goat IgG (H + L) cross-adsorbed secondary antibody), and DRAQ5™ fluorescent probe solution were obtained from Thermo Fisher Scientific.

Total RNAs from healthy human lung, brain, small intestine, colon, and placenta were purchased from Thermo Fisher Scientific. Human Lung Whole Tissue Lysate (Adult Whole Normal) was obtained from Novus Biologicals and protein extracts from healthy human small intestine and colon were obtained from Santa Cruz Biotechnology. Lysate from HEK293T cells overexpressing full-length RAGE and a lysate from mock-transfected HEK293T cells were purchased from Novus Biological.

### 4.2 Biological material

Placentas were obtained from healthy women as well as mothers suffering from GDM, PE or FGR (n = 3–4 per group) after cesarian section at 23–39 weeks of gestation at the Vienna General Hospital (Table 2). The study was conducted in accordance with the Declaration of Helsinki. All study participants gave written informed consent and the study was approved by the Ethics Committee of Medical University of Vienna/AKH, EC No. 1035/2015. Placentas were immediately processed after cesarian section to minimize destructive processes. For analysis of protein expression by WB and ELISA assay, the tissue was dissected, small pieces were snap frozen in liquid nitrogen (N_2_) and stored at −80 °C. Total protein lysates from placental tissue were prepared in T-PER buffer (1 mL per 50 mg crushed tissue; Thermo Fisher Scientific) according to the protocol of the manufacturer and protease inhibitor cocktail (Halt™ Protease Inhibitor Cocktail, Thermo Fisher Scientific) was added. The method for fixation, embedding, sectioning and immunofluorescence staining of placental tissue is described below in the immunofluorescence staining section.

**Table 2:** Characteristics of women, babies and placentas.

Diagnosis	Sample	Maternal age	Week of pregnancy	Placental weight (g)	Sex of baby
**Healthy**	H1	35	39 + 4	650	f
	H2	30	38 + 4	546	m
	H3	35	38 + 5	522	f
**GDM**	GDM1	30	38 + 6	750	f
	GDM2	36	38 + 1	378	m
	GDM3	38	39 + 1	667	f
**PE**	PE1	27	23 + 5	120	m
	PE2	40	33 + 0	334	f
	PE3	41	33 + 2	408	f
**FGR**	FGR1	39	38 + 0	404	m
	FGR2	32	32 + 1	200	f
	FGR3	28	27 + 0	149	f
	FGR4	29	32 + 5	286	m

GDM, gestational diabetes mellitus; PE, preeclampsia; FGR, fetal growth restriction.

### 4.3 Generation of full-length RAGE expressing cells

The sequence coding for full-length RAGE (UniProt Q15109) was cloned into a retroviral expression vector and stably expressed in the Jurkat (JE6.1) cell line. Surface expression was analyzed by flow cytometry using anti-RAGE MAB11451 (R&D Systems). Bound antibodies were detected with goat anti-mouse IgGFcγ-APC (Jackson ImmunoResearch).

### 4.4 Cell culture

All cell culture media were supplemented with 10% fetal bovine serum (FBS), penicillin, and streptomycin unless otherwise stated. Jurkat cells (JE6-1) were cultured in RPMI 1640 medium (GlutaMAX; Gibco). Caco-2 (ATCC) cells – epithelial cells derived from a colon adenocarcinoma – were cultured in Dulbecco's modified Eagle's medium (DMEM, GlutaMAX, high-glucose; Gibco) supplemented with 1% non-essential amino acids and FHs 74 Int cells (ATCC) – epithelial cells derived from healthy fetal small intestine – were cultured in Hybri-Care medium (ATCC) supplemented with 30 ng/mL epidermal growth factor (Biozym). BeWo cells were cultured in Dulbecco's modified Eagle's medium (DMEM, GlutaMAX, high-glucose; Gibco). All cell lines were cultivated at 37 °C in a humidified atmosphere (95%) containing 5% CO_2_. Cultured cells were washed with PBS and lysed in cell lysis buffer (150 mM NaCl, 50 mM Tris pH8, 1% Triton X-100) containing protease inhibitor (Roche). Protein concentrations were determined by Pierce™ BCA Protein Assay Kit (Thermo Fisher Scientific).

### 4.5 Analysis of splice variants

One microgram of total tissue RNA was used for oligo-dT primed cDNA synthesis using the First Strand cDNA Synthesis Kit (Thermo Fisher Scientific). RAGE splice isoforms were amplified from cDNA using a nested PCR approach. The primary PCR was performed with primers 5′UTR-F and 3′UTR-R (Table 3, Fig. 1). The resulting PCR product was diluted 1:10 and used for a second PCR using primers Ex1-F and Ex11-R. The final products were separated by agarose gel electrophoresis, bands from 1000 to 1500 bp were excised, purified using the Monarch Gel Extraction Kit (New England BioLabs) and cloned into the pGEM-Teasy Vector (Promega). Bacterial clones were picked for colony PCR using primers Ex1-F and Ex6-R as well as Ex6-F and Ex11-R2. Splice variants were identified by gel electrophoresis based on PCR product size and verified by sequencing. For sequencing, plasmid DNA was purified using the NucleoSpin Plasmid Miniprep Kit (Macherey-Nagel). All PCRs were performed using the DreamTaq Green PCR Master Mix (Thermo Fisher Scientific). The sequence data have been submitted to GenBank and correspond to the accession numbers: ON814573 (RAGE_vKS1), ON814574 (RAGE_vKS2), ON814575 (RAGE_vKS3), ON814576 (RAGE_vKS4), and ON814577 (RAGE_vKS5).

**Table 3:** RAGE primers.

Name	Primer Sequence	Previous name	Ref.
5′-UTR-F	CAGGACCCTGGAAGGAAGCAGG	RAGE_extF	[20]
Ex1-F	GCCGGAACAGCAGTTGGAGCC	RAGE_intF	[20]
Ex6-F	GATCCCCGTCCCACCTTCTCCTGTAGC	RAGE up	[84]
Ex6-R	GCTACAGGAGAAGGTGGGACGGGGATC		
Ex11-R	GCCCTCCAGTACTACTCTCGCC	RAGE_intR	[20]
Ex11-R2	CACGCTCCTCCTCTTCCTCCTGGTTTTCTG	RAGE lo	[84]
3′-UTR-R	CTGGTTGTAGAAGAAAGCTTGGC	RAGE_extR	[20]

### 4.6 RAGE expression analysis

The GENEVESTIGATOR platform (NEBION AG, Zurich, Switzerland), which integrates publicly available and manually curated microarray and RNA sequencing data sets, was used to extract RAGE expression levels [50].

### 4.7 Immunoblotting

Placenta and HEK293 lysates were precipitated with acetone on ice and protein pellets were dissolved in 1× reducing SDS–PAGE sample buffer. N-linked oligosaccharides were removed enzymatically using PNGase F (New England Biolabs) according to the manufacturers’ instructions. Lysates were separated by 10% reducing SDS-PAGE and transferred to PVDF membranes (GE Healthcare). After blocking with 5% (w/v) milk powder in TBS 0.1% (v/v) Tween (TBST) over-night, membranes were incubated with the respective primary antibody at a concentration of 0.4 μg/mL (SC A11 and AF1145), and 1 μg/mL (AB D01P and MAB11451) for 3 h at room temperature or over-night at 4 °C. Membranes were washed with TBST, incubated with the respective HRP-conjugated secondary antibody for 1 h at room temperature and immunoreactivity was visualized using chemoluminescence. For a better estimation of molecular masses and to provide information on any additional reactive signal, we show the entire blots [51] as well as control blots lacking the primary antibody.

### 4.8 ELISA

A human RAGE-specific ELISA kit (RK00844) was purchased from ABclonal. Undiluted lysates from healthy, GDM, PE and FGR (n = 3 per group) placentas as well as human lung whole tissue lysate were processed according to the kit instructions. Amount of RAGE (pg) per sample volume was calculated using the provided internal RAGE standard in a concentration range from 0 to 2000 pg/mL. The results were corrected for the protein concentration in the respective samples and are displayed as pg RAGE/mg protein (Table 1).

### 4.9 Immunofluorescence staining and confocal microscopy

Adherent cells were grown in chamber slides (μ-Slide 8 well, ibiTreat, tissue culture treated, Ibidi GmbH) until reaching confluency and fixed with Image-iT™ Fixative Solution (FB002; 4% formaldehyde, methanol-free) for 15 min. Suspension cells were treated according to a protocol published by Tsang and colleagues [85]. Briefly, cells were pelleted by centrifugation, washed with phosphate buffered saline (PBS) and resuspended in PBS at a concentration of 1 × 10^6^ cells/mL. Cells were transferred to a chamber slide and left at room temperature to allow for sedimentation and adhesion for 30 min. After careful removal of non-adherent cells, cells were fixed. For blocking and permeabilization cells were treated with PBS containing 5% (v/v) goat or donkey serum (Sigma-Aldrich) and 0.05% (w/v) saponin (Sigma-Aldrich). After washing, the cells were stained with RAGE-specific antibodies at a concentration of 4 μg/mL (Fig. 8A) and μg/mL (Fig. 8B) (SC A11), 4 μg/mL (AF1145), and 10 μg/mL (AB D01P and MAB11451) and detection was performed with the respective AlexaFluor-568-conjugated secondary antibodies. Nuclei were counterstained with DRAQ5 and after a final washing step cells were overlaid with PBS. Confocal images were acquired using an UltraVIEW ERS Rapid Confocal Imager (PerkinElmer) connected to a Zeiss Axiovert 200 microscope fitted with a 63×/1.4 oil objective lens (Plan-Apochromat, Zeiss). The fluorophores were excited using a 488/548/647 multiline argon/krypton laser. Pictures were digitalized and processed by Volocity software (Version 5.5, PerkinElmer). Files were exported and further processed with ImageJ2 (Version 2.3.0) using identical conditions for positive and negative controls.

For analysis of placental sections, chorionic tissue was processed by HOPE-fixation (Hepes-Glutamic acid buffer mediated Organic solvent Protection Effect; DCS Innovative Diagnostik-Systeme) and paraffin-embedding [86,87]. HOPE fixation is a potent alternative to conventional formalin-fixation and cryo-techniques, and combines the benefits of both, i.e., morphological preservation and antigen detection with cryo-compatible antibodies. Tissue sections (4 μm) were de-waxed and re-hydrated [88]. Antigen retrieval was done with 0.05% (v/v) citraconic anhydride solution, pH 7.4, for 20 min [89]. Sections were incubated with blocking buffer (5% (v/v) goat or donkey serum in PBS containing 0.05% (w/v)) saponin for 1 h at room temperature. Primary anti-RAGE antibodies were all diluted 1:20 in blocking buffer and antibodies against cell markers (CD163, CK7 and CD31) were all diluted 1:100 in blocking buffer. Primary and secondary antibodies were applied overnight at 4 °C or for 2 h at room temperature, respectively. In negative control incubations, primary antibodies were omitted. Nuclei were stained with 4′,6-diamidino-2-phenylindole, dihydrochloride (DAPI; Roche Diagnostics GmbH; 50 μg/mL in PBS). After each incubation step, sections were washed intensively with PBS. Fluoromount-G™ mounting medium (Thermo Fisher Scientific) was used as mounting medium. Images were acquired using an automated widefield fluorescence microscope (Axio Imager Z1, Zeiss), equipped with an EC Plan-Neofluar 20×/0.5 objective (Plan-Neofluar, Zeiss) and the following filter sets (Chroma Technology Corp.): 49000 ET-DAPI, 49008 ET-mCherry, and TxRed, in combination with TissueFAXS Image Acquisition and Management Software (Version 6.0; TissueGnostics GmbH). Using a monochrome camera (Hamamatsu), grayscale images of individual fluorescence channels were acquired. Acquired regions were composed of at least 5 × 5 single images. Pseudo-colors were assigned to the individual images and selected fluorescence channels were combined and exported. Files were further processed with Adobe Photoshop (Version Elements 2020) using identical conditions for positive and negative controls.

## Declarations

All study participants gave written informed consent and the study was approved by the Ethics Committee of Medical University of Vienna/AKH, EC No. 1035/2015.

## Author contributions

KS, IE, and SG conceived and designed the experiments. KS, KG, JL, RV, IE and SG performed the experiments. KS, IE, and SG analyzed and interpreted the data. PS, CG, KHS, IE, and SG contributed reagents, materials, analysis tools or data. KS, IE, and SG wrote the paper. All authors revised the manuscript and approved the submitted version.

## Declaration of competing interest

The authors declare that they have no known competing financial interests or personal relationships that could have appeared to influence the work reported in this paper.
