# Supplementary material for: Human intestine and placenta exhibit tissue-specific expression of RAGE isoforms
Source: Heliyon. 2023 Jul 18;9(8):e18247. doi: 10.1016/j.heliyon.2023.e18247 (PMC10391957; doi:10.1016/j.heliyon.2023.e18247)
Supplement: Multimedia component 1 [file mmc1.pdf]

SUPPLEMENTARY FIGURES

Human intestine and placenta exhibit tissue-specific expression of RAGE isoforms

Katharina Schwertner[1], Katharina Gelles[1], Judith Leitner[2], Peter Steinberger[2], Claudia Gundacker[3], Ruben Vrticka[1], Karin Hoffmann-Sommergruber[1], Isabella Ellinger[1,*], and Sabine Geiselhart[1,*]

[1] Institute of Pathophysiology and Allergy Research, Medical University of Vienna, Vienna, Austria

[2] Institute of Immunology, Medical University of Vienna, Vienna, Austria

[3] Institute of Medical Genetics, Medical University of Vienna, Vienna, Austria

* Correspondence: sabine.geiselhart@muv.ac.at; isabella.ellinger@meduniwien.ac.at

Figure S1

A

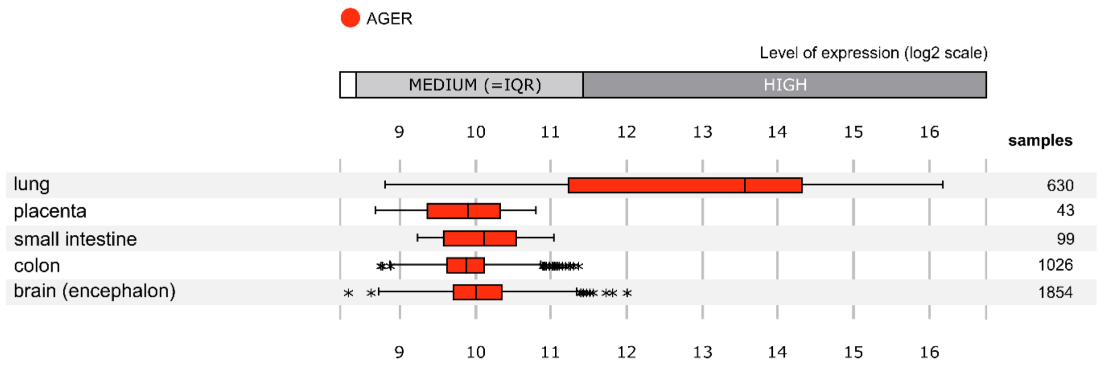

B

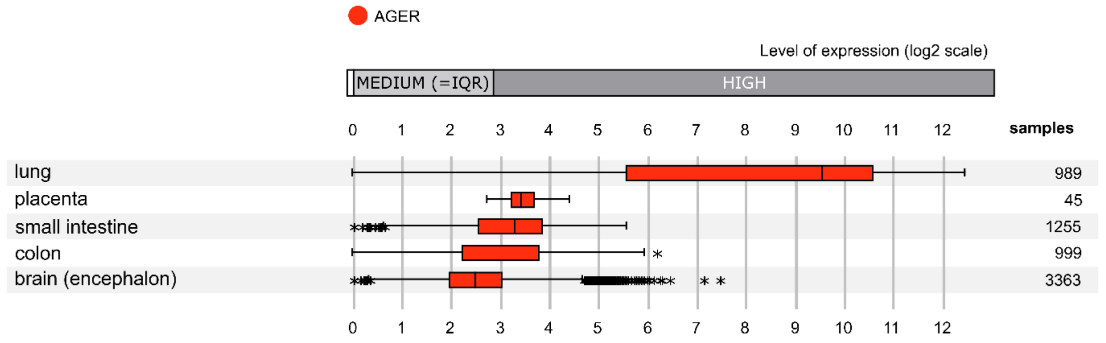

Figure S1. GENEVESTIGATOR-based expression profile of the RAGE gene (AGER) of publically available and curated microarray data sets from the Affymetrix Human Genome U133 Plus 2.0 Array platform (**A**) or mRNA-Seq Gene Level Homo sapiens (Ref. Ensembl 97, GRCh38) platform (**B**). Expression profiles are illustrated in the corresponding boxplot.

Figure S2

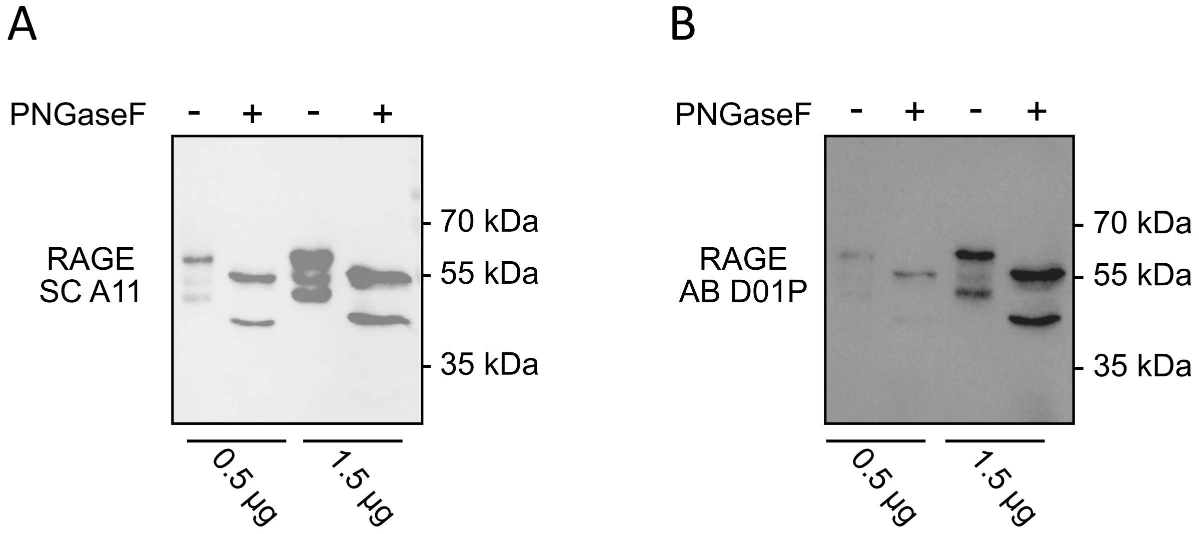

Figure S2. RAGE immunoblotting analysis of non-deglycosylated and deglycosylated human lung lysate. SDS-PAGE was performed under reducing conditions. Membranes were stained with two RAGE-specific antibodies; SC A11 (A), and AB D01P (B). The uncropped images are shown in the supplementary material of the manuscript.

Figure S3

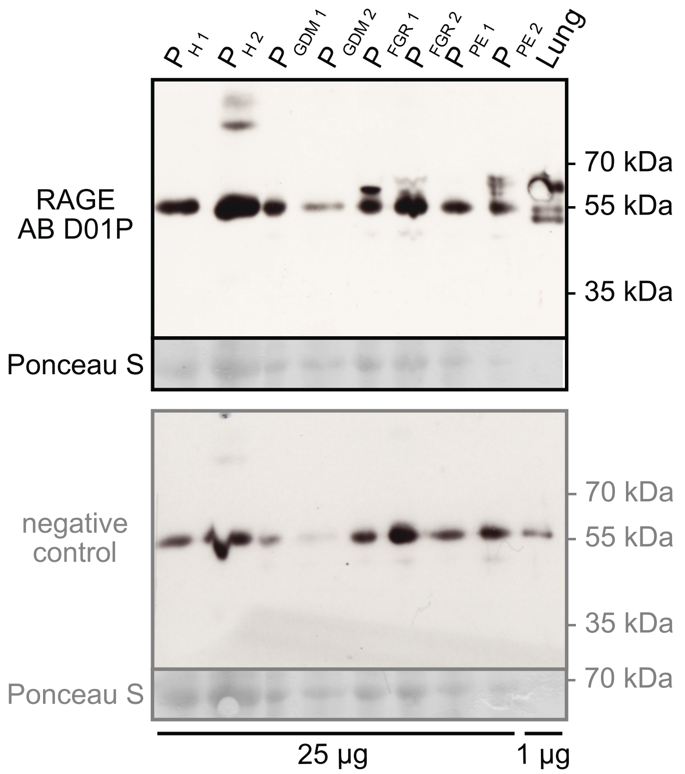

Figure S3. RAGE immunoblotting analysis of human placenta and lung tissue. SDS-PAGE was performed under reducing conditions. The membranes were stained with the RAGE-specific AB D01P antibody or with the secondary antibody only (negative control). The uncropped images are shown in the supplementary material of the manuscript.
