# Supplementary material for: Human intestine and placenta exhibit tissue-specific expression of RAGE isoforms
Source: Heliyon. 2023 Jul 18;9(8):e18247. doi: 10.1016/j.heliyon.2023.e18247 (PMC10391957; doi:10.1016/j.heliyon.2023.e18247)
Supplement: Multimedia component 2 [file mmc2.pdf]

Supplementary Material - Uncropped images

Figure 1B

PCR #1

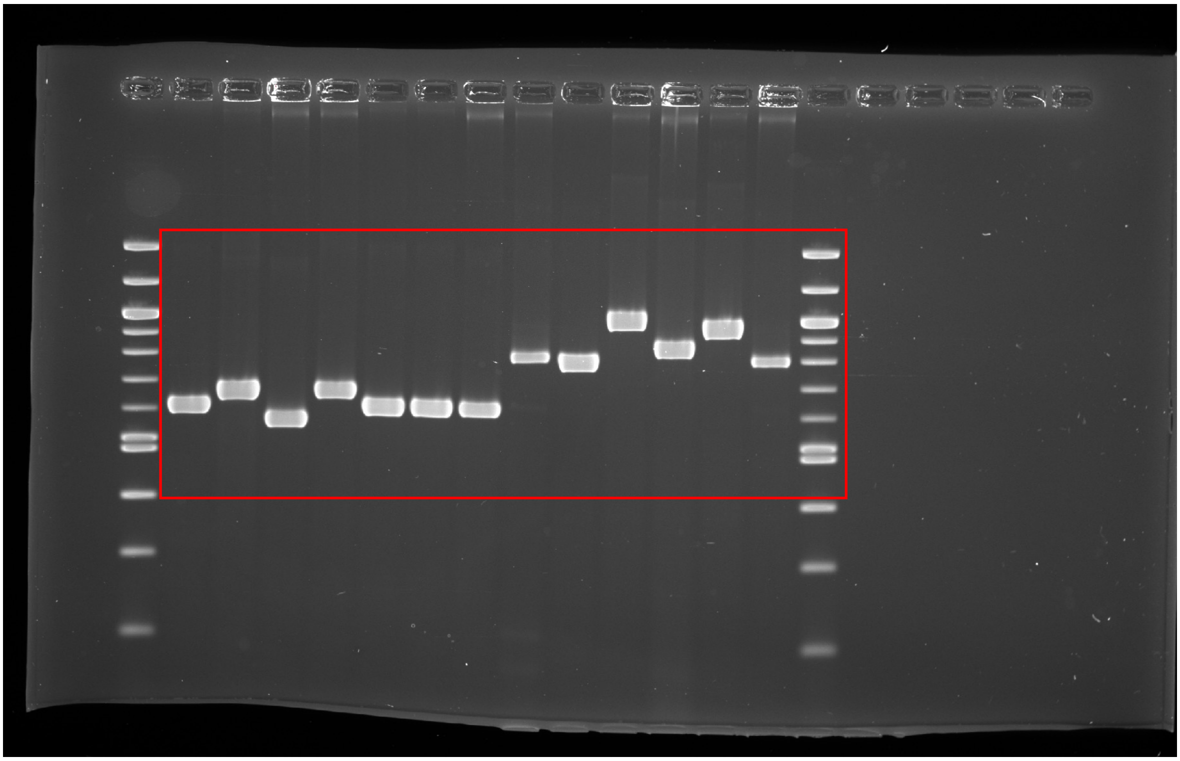

PCR #2

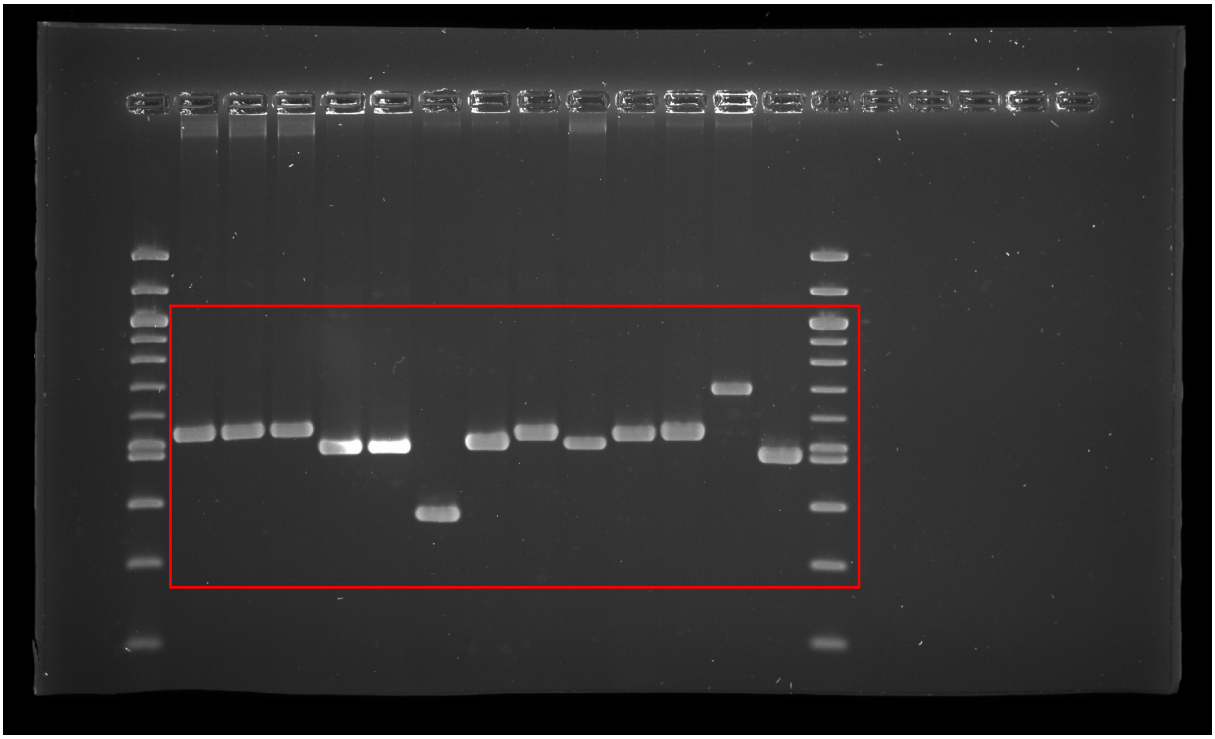

Figure 4A

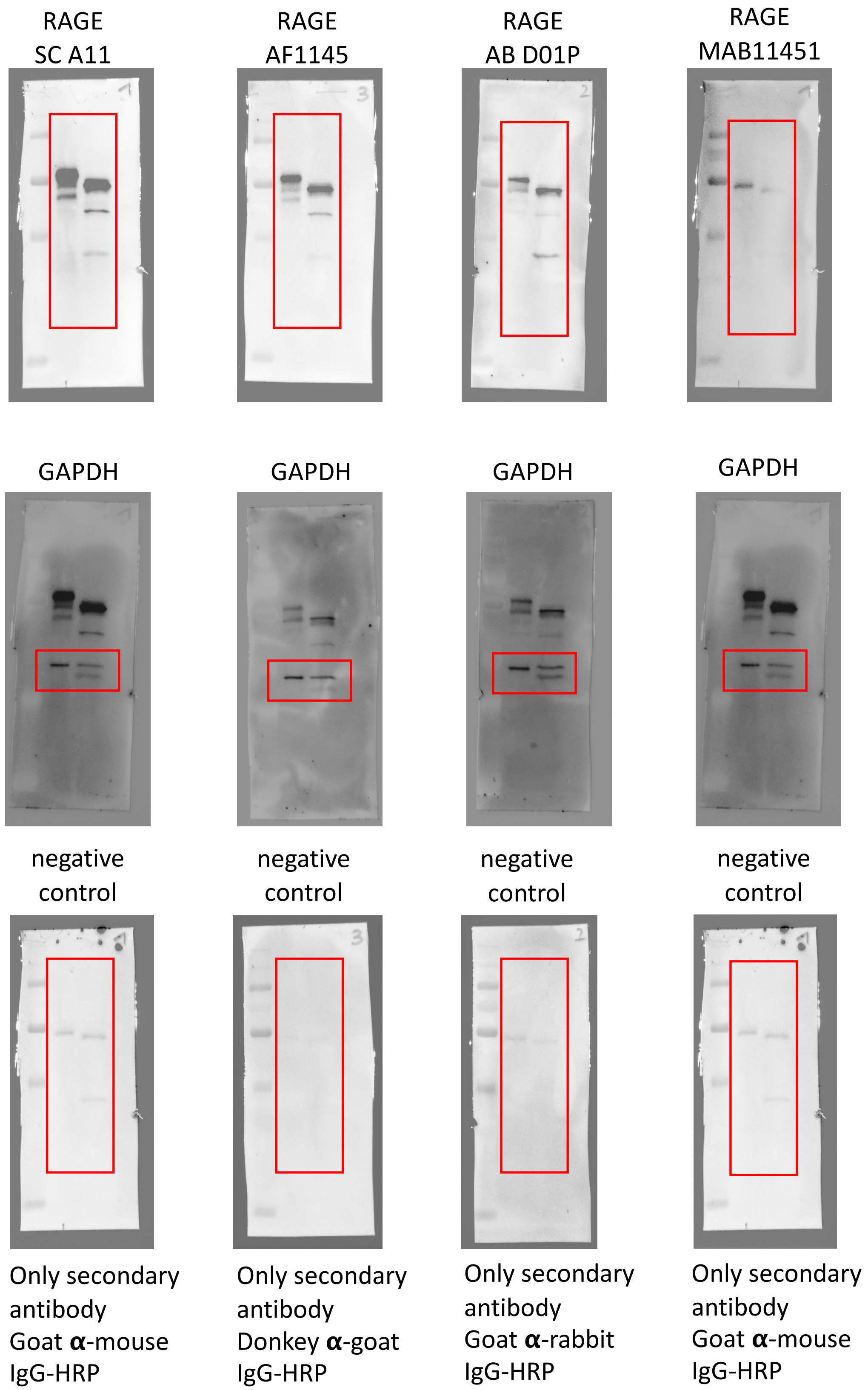

Loading scheme for all membranes:

Protein ladder / Lung lysate / PNGase F-treated lung lysate

Three membranes were prepared; membranes shown in column 1 and 4 are the same membrane. The membranes were first incubated with the secondary antibody alone (results are shown in the third row). Then, the membranes were incubated with the indicated anti-RAGE antibody plus corresponding secondary antibody. Note that the membrane incubated with RAGE MAB11451 was subsequently incubated with RAGE SC A11 (first row). Then, the membranes were incubated with the anti-GAPDH antibody and a corresponding secondary antibody to visualize the amount of samples loaded (second row).

Figure 4B

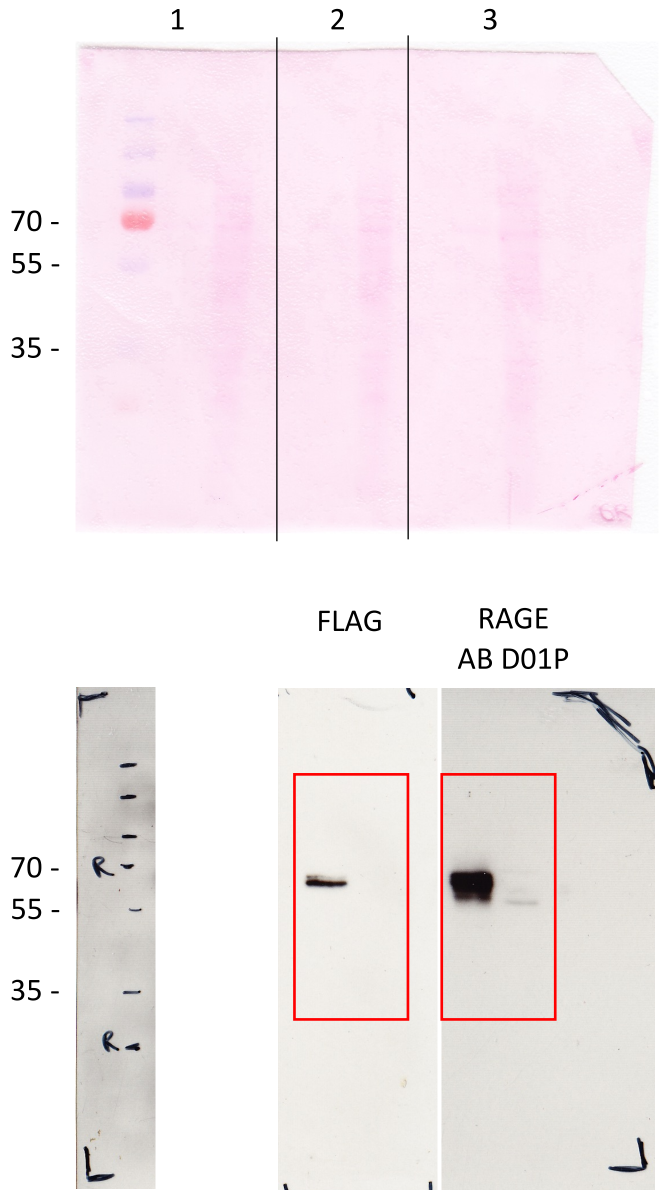

Loading scheme:
Protein ladder/ lysate from HEK293T cells overexpressing full-length RAGE / lysate from mock-transfected HEK293T cells /sample buffer / lysate from HEK293T cells overexpressing full-length RAGE / lysate from mock-transfected HEK293T cells /sample buffer / lysate from HEK293T cells overexpressing full-length RAGE / lysate from mock-transfected HEK293T cells / sample buffer.
Identical volumes (1 uL) of the two different lysates were loaded.

Following Ponceau S staining, the membrane was cut in three parts as indicated by the black lines.

Part 2 and 3 of the membrane were used to generate Figure 4B. Part 2 was incubated with the ani-FLAG antibody and a corresponding secondary antibody, while part 3 was incubated with the indicated anti-RAGE antibody plus the corresponding secondary antibody.

Figure 5

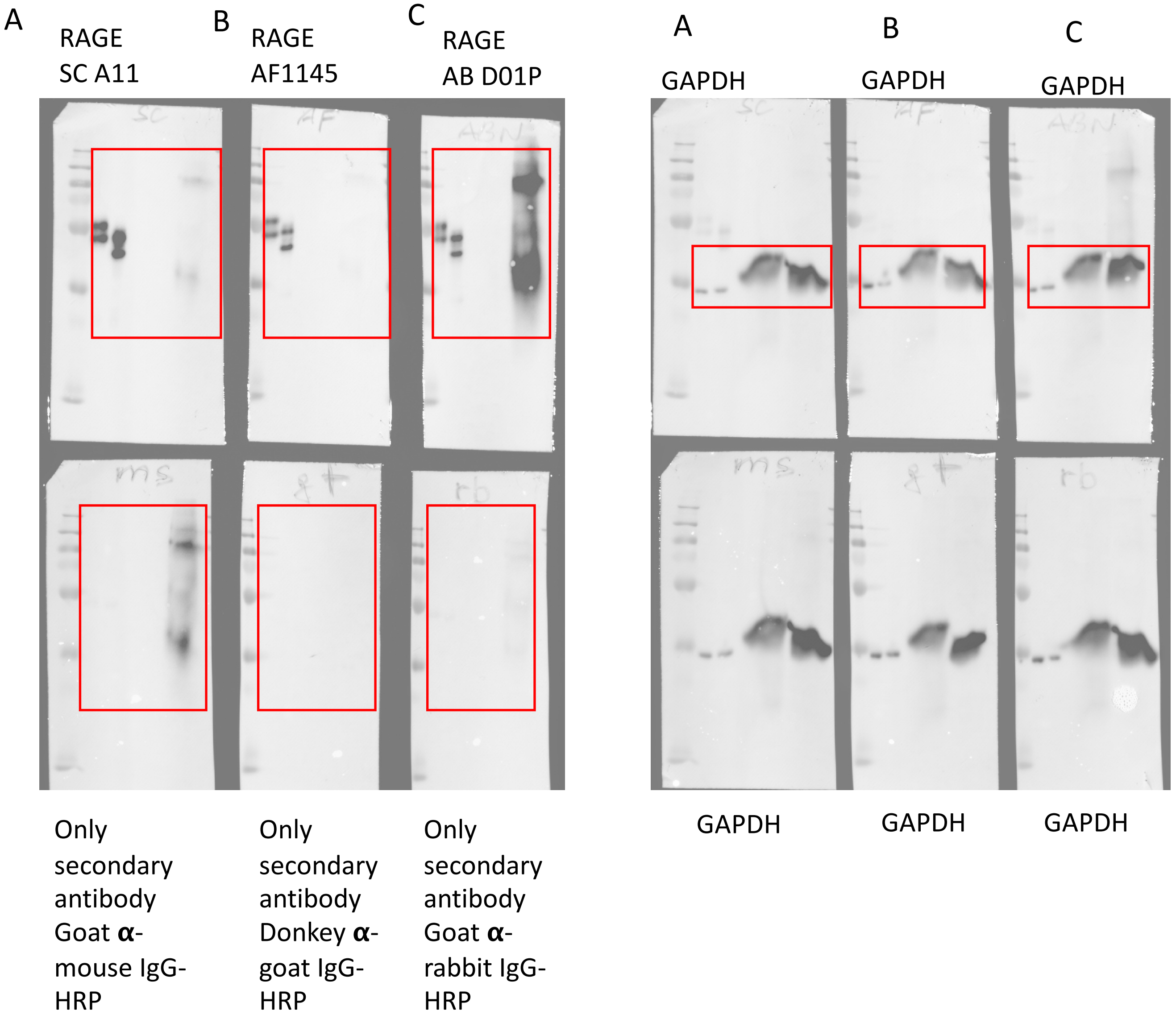

Loading scheme for all membranes
Protein ladder / Lung lysate / PNGase F-treated lung lysate / SI lysate / Colon lysate

Six identical membranes were prepared; three membranes were incubated with the indicated anti-RAGE antibody plus corresponding secondary antibody (A, B, C first row, left side). The other three membranes were only incubated with the secondary antibodies alone (second row, left side). Then, the membranes were incubated with the anti-GAPDH antibody and a corresponding secondary antibody to visualize the amount of sample loaded (right side).

Figure 6

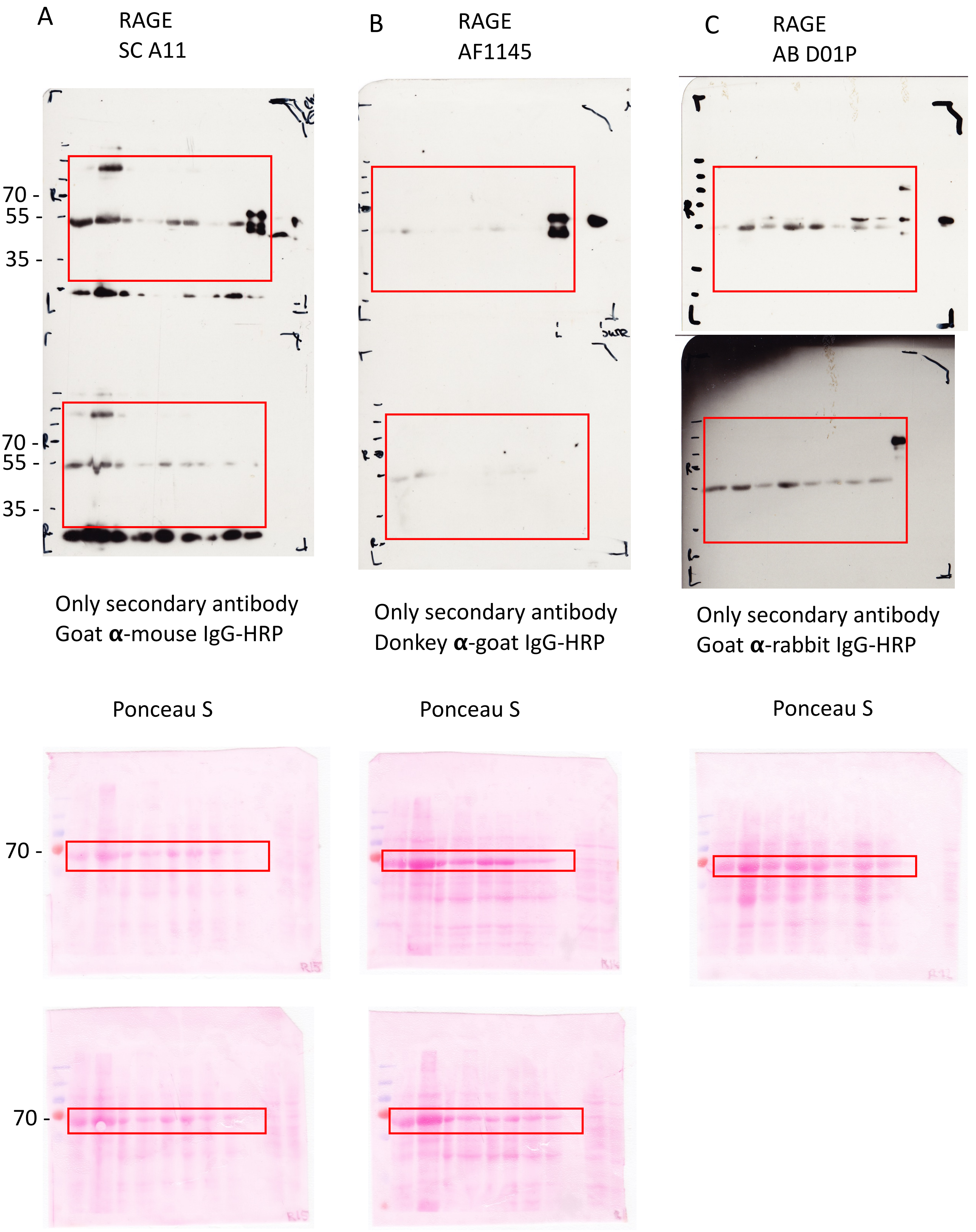

Loading scheme for all membranes

Protein ladder / Eight different placental lysates / Lung lysate / 1-2 additional samples not included in the figure.

For A and B, two identical membranes were prepared. One membrane was incubated with the indicated anti-RAGE antibody plus corresponding secondary antibody (A, B first row). The second membrane was only incubated with the secondary antibody alone (A, B second row). Ponceau S stained membranes are shown below. For C, one membrane was first incubated with the secondary antibody alone (C, second row) and thereafter with the indicated anti-RAGE antibody plus corresponding secondary antibody (C, first row). Ponceau S stained membrane is shown below.

Figure 7

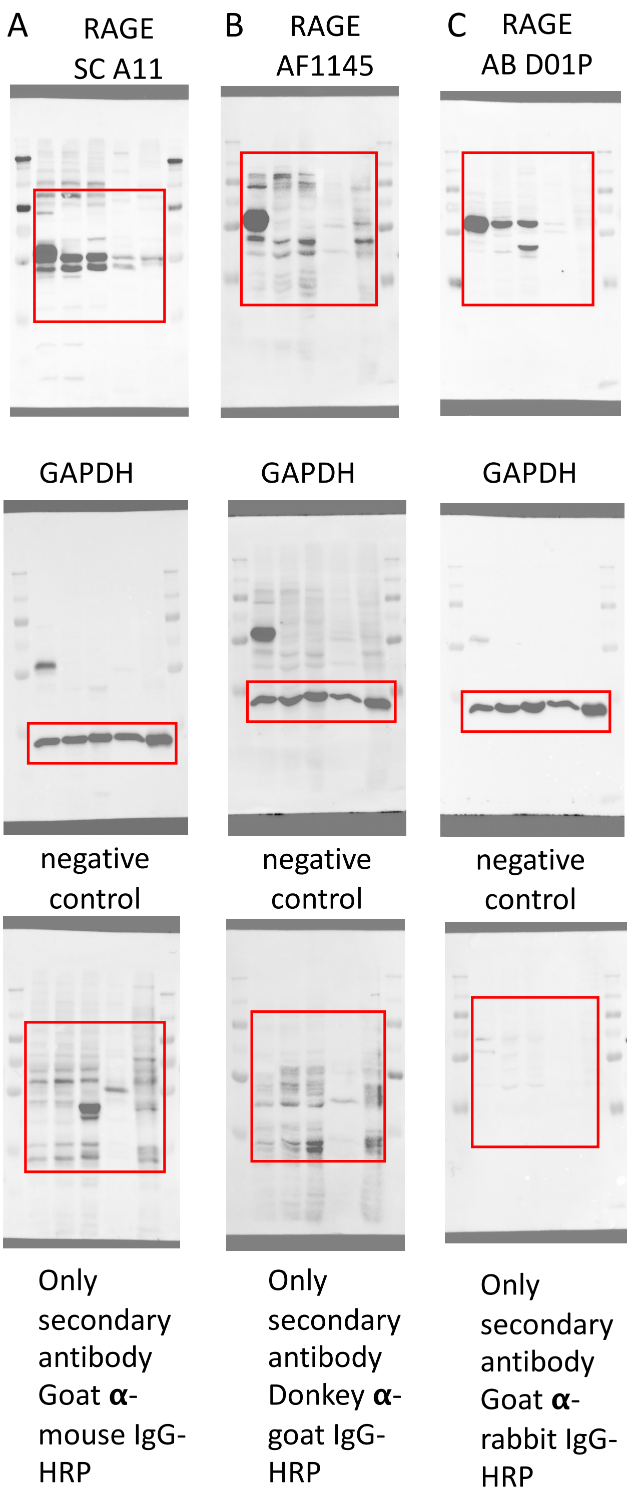

Loading scheme:
Protein ladder / JE6-1 RAGE / JE6-1 / Caco-2 / FHs 74 Int / BeWo / Protein ladder

Three membranes were prepared. The membranes were first incubated with the secondary antibody alone (results are shown in the third row). Then, the membranes were incubated with the indicated anti-RAGE antibody plus corresponding secondary antibody. Then, the membranes were incubated with the anti-GAPDH antibody and a corresponding secondary antibody to visualize the amount loaded (second row).

Figure S2

A RAGE SC A11

B RAGE AB D01P

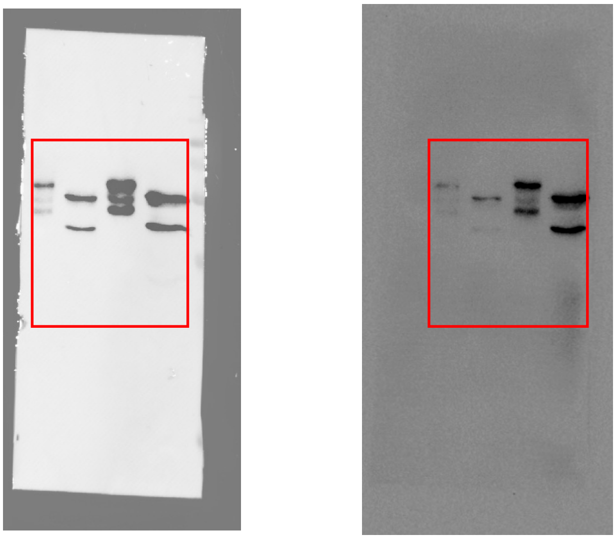

Loading scheme:

Lung lysate / PNGase F-treated lung lysate / Lung lysate / PNGase F-treated lung lysate / Protein ladder

0.5 µg 1.5 µg

Figure S3

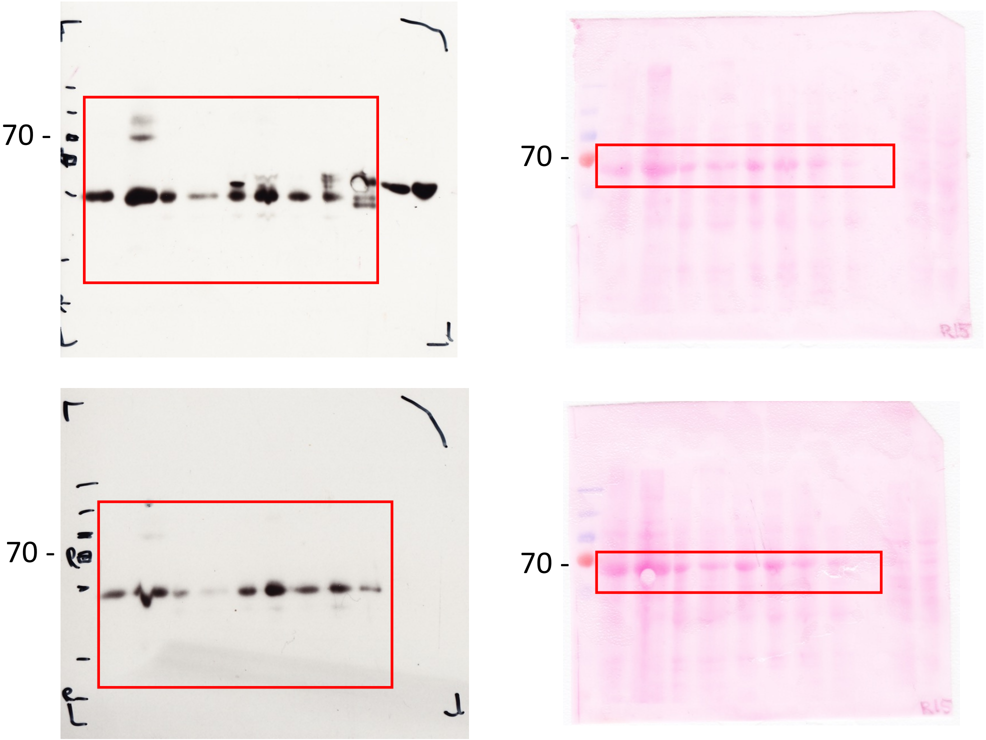

RAGE
AB D01P

Only secondary antibody
Goat α-rabbit IgG-HRP

Loading scheme

Protein ladder / Eight different placental lysates / Lung lysate / Two additional samples not included in the figure

Two identical membranes were prepared. One membrane was incubated with the indicated anti-RAGE antibody plus corresponding secondary antibody (first row). The second membrane was only incubated with the secondary antibody alone (second row). Ponceau S stained membranes are shown next to the blots.
